# Supplementary material for: Network integration of thermal proteome profiling with multi-omics data decodes PARP inhibition
Source: Mol Syst Biol. 2024 Mar 7;20(4):10. doi: 10.1038/s44320-024-00025-w (PMC10987601; doi:10.1038/s44320-024-00025-w)
Supplement: Supplementary file 8 — Expanded View Figures [file 44320_2024_25_MOESM8_ESM.pdf]

Expanded View Figures

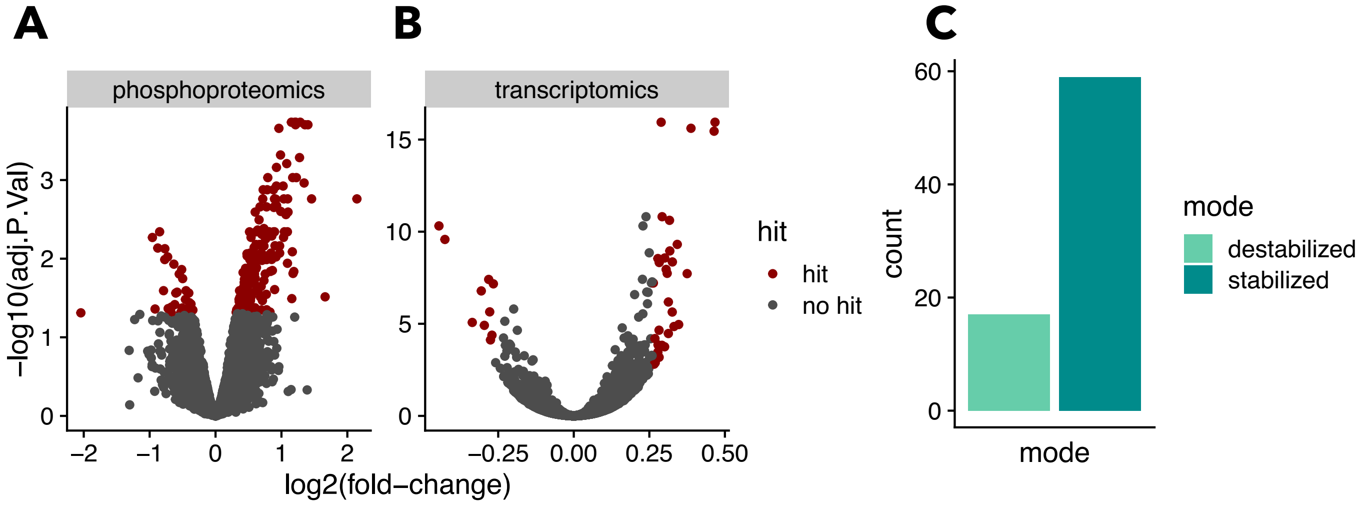

Figure EV1. Transcriptomics, phosphoproteomics, and TPP data.

UWB1.289 cells were treated with 4 uM Olaparib for 24 h to generate direct and indirect treatment effects before multiple omics datasets were acquired. (**A**) Volcano plot of the phosphoproteomics data after limma analysis comparing Olaparib treatment to a DMSO control ($n = 11610$ phosphosites, adjusted *p*.value <0.05, *t*-test). (**B**) Volcano plot of the transcriptomics data after limma analysis comparing Olaparib treatment to a DMSO control ($n = 13{,}710$ transcripts, adjusted *p*.value <0.05, *t*-test). (**C**) Overview of the Olaparib TPP hits determined using the F-statistic-based hit calling approach.

© The Author(s)

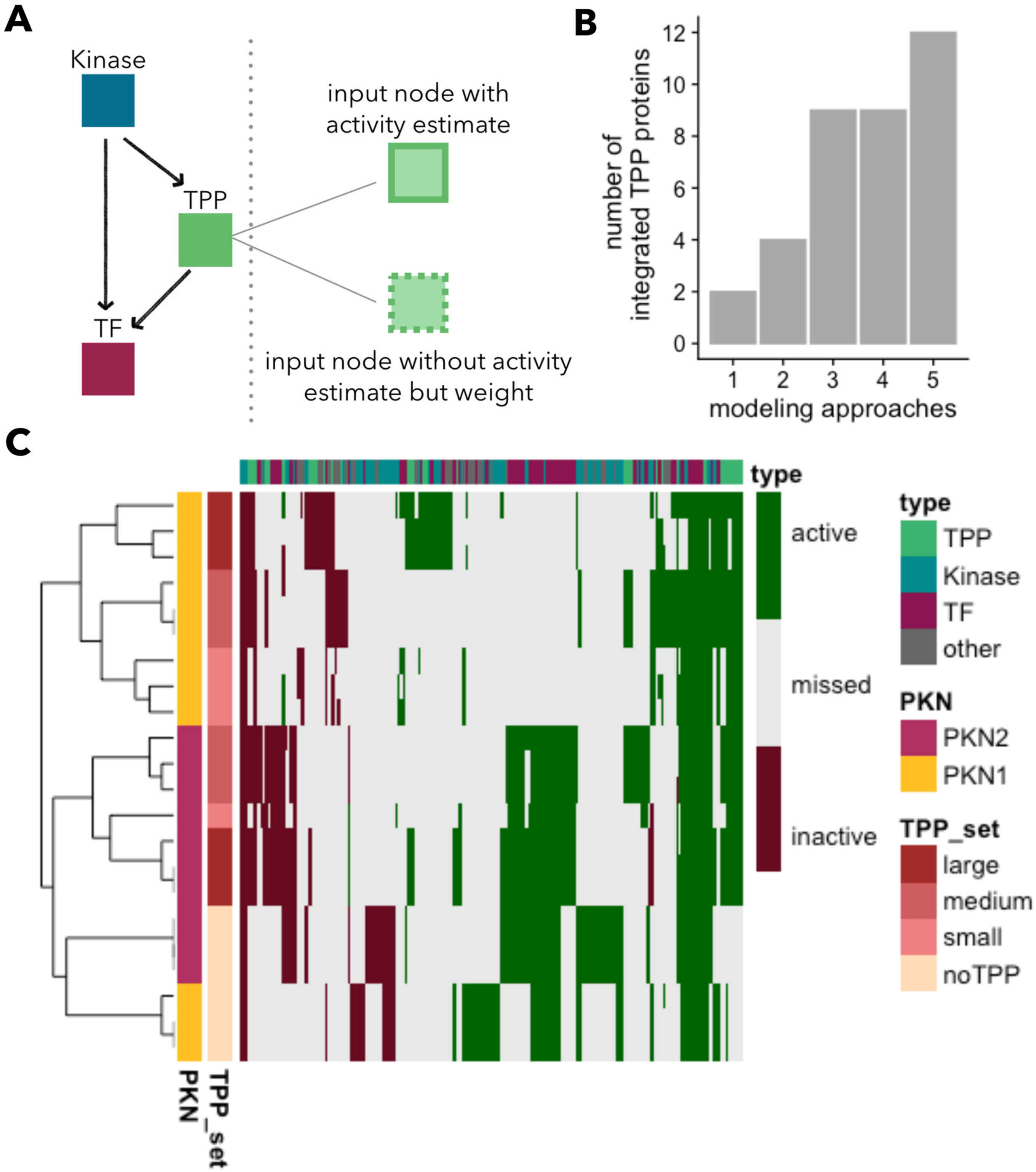
A
Kinase
TPP
TF
input node with
activity estimate
input node without activity
estimate but weight
B
number of
integrated TPP proteins
modeling approaches
C
type
active
missed
inactive
type
TPP
Kinase
TF
other
PKN
PKN2
PKN1
TPP_set
large
medium
small
noTPP
PKN
TPP_set

 © The Author(s)

Figure EV2. Robustness and reproducibility.

We tried to validate whether the chosen setup (combination of runs, activity estimation and filtering steps) can be used to model reasonable networks with COSMOS. (**A**) We integrated TPP proteins between upstream kinases and downstream transcription factors into the signaling cascade based on the correlation of phosphoproteomic and TPP data. Further, we implemented a strategy for activity estimation where we inferred activities for TPP proteins via upstream kinases and phosphorylation status. For TPP proteins without activity estimate, we inferred the activity state during the optimization supported by a weight reflecting (de)stabilization. (**B**) Different modeling setups (1–5) were tested to maximize the number of integrated TPP proteins which we reached using activity estimations and weights. 1: no TPP proteins as input. 2: TPP proteins without activity information. 3: TPP with activity estimation. 4: TPP with activity estimation and weights. 5: TPP with activity estimation, NA values and weights. Running the optimization without TPP data still yields few TPP proteins in the solution, due to overlaps with other input modalities or the inclusion of prior knowledge. Details of the optimization process are provided in the CARNIVAL and COSMOS publications (Liu et al, 2019; Dugourd et al, 2021). (**C**) In principle, the integer linear programming solver optimizes towards a local optimum and the complete search space of the problem is not known. As a consequence, multiple technical replicates of COSMOS can have similar but slightly different optimal solutions. To assess if this solver-dependent variability can be distinguished from actual differences due to the used input data, we set up a small robustness analysis. We clustered replicate networks for different TPP set sizes based on node activities (active, missed, inactive). All replicate runs of distinct TPP sets cluster together. The networks separate into networks with TPP input and networks without TPP input. Further the influence of the used prior knowledge is clearly visible (PKN).

© The Author(s)
